# Development and Validation of a Bioanalytical Method for 3′- and 6′-Sialyllactose in Minipig Liver and Kidney Using Liquid Chromatography-Tandem Mass Spectrometry and Its Application to Analysis of Tissue Distribution

**DOI:** 10.3390/molecules25235721

**Published:** 2020-12-03

**Authors:** Han Young Eom, Seok-In Jang, Jong-Hwa Lee

**Affiliations:** Bioanalysis and Pharmacokinetic Study Group, Korea Institute of Toxicology, Daejeon 34114, Korea; hanyoung.eom@kitox.re.kr (H.Y.E.); sijang@kitox.re.kr (S.-I.J.)

**Keywords:** sialyllactose, LC-MS/MS, minipig, method validation, tissue

## Abstract

Breast milk contains human milk oligosaccharides (HMOs), including sialyllactose (SL). SL is composed of sialic acid and lactose, and is divided into 3′-SL and 6′-SL according to the binding position. SL has immunoprotective effects against bacteria and viruses, and acts as a probiotic in the gastrointestinal tract. In this study, we developed a bioanalytical method for simultaneous analysis of 3′-SL and 6′-SL in liver and kidney tissues of Yucatan minipigs using liquid chromatography–tandem mass spectrometry (LC-MS/MS) under conditions optimized in our previous study. LC-MS/MS was performed using a hydrophilic interaction liquid chromatography (HILIC) column (50 mm × 2.1 mm, 3 μm) with a mobile phase consisting of 10 mM ammonium acetate in water (pH 4.5) and acetonitrile with gradient elution at a flow rate of 0.3 mL/min. A surrogate matrix method using water was applied for analysis of endogenous SL. The developed method was validated with regard to selectivity, linearity, precision, accuracy, the matrix effect, recovery, parallelism, dilution integrity, carryover, and stability according to the US Food and Drug Administration guidelines. We performed a tissue distribution study of minipigs, and analyzed liver and kidney tissues using the developed method to determine the tissue distribution of 3′-SL and 6′-SL. The tissue concentrations of 3′-SL and 6′-SL were readily measurable, suggesting that the method would be useful for evaluating the tissue distributions of these compounds in minipigs.

## 1. Introduction

The major sialyloligosaccharides in breast milk are 3′- and 6′-sialyllactose (3′-SL and 6′-SL, respectively), disialyllacto-*N*-tetraose (DSLNT), and sialyllacto-*N*-tetraose (SLNT). 3′-SL and 6′-SL are endogenous compounds within the body, and the level of 3′-SL is maintained during lactation, but 6′-SL level decreases gradually in breast milk over time [1]. SL has an antiinflammatory effect that inhibits bacterial adhesion in the gastrointestinal tract and acts as a potential inhibitor of enterotoxins [2,3]. In addition, SL has been reported to reduce infectivity of influenza virus, human immunodeficiency virus, and rotavirus by acting as a molecular marker of the capture of mature dendritic cells, and also suppresses cholera toxin [4,5]. SL acts as a probiotic by supporting the growth of beneficial intestinal bacteria belonging to the genera *Bifidobacterium* and *Lactobacillus* [6,7,8].

Previous studies analyzed human milk oligosaccharides (HMOs) in milk and biological matrices using various methods, including high-performance liquid chromatography–ultraviolet detection (HPLC-UV) [9], high-performance anion-exchange chromatography with pulsed amperometric detection [10,11], capillary electrophoresis (CE) [12], matrix-assisted laser desorption/ionization time-of-flight mass spectrometry (MALDI-TOF MS) [13,14,15], MALDI-Fourier transform ion cyclotron resonance mass spectrometry (MALDI-FTICR MS) [16], electrospray ionization mass spectrometry (ESI-MS) [17], and microfluidic chip–time-of-flight mass spectrometry (HPLC-Chip/TOF MS) [18,19]. HMO does not have a strong chromophore, so UV detection has low sensitivity for its detection. Moreover, because of its high polarity, it is not retained well in general reversed-phase columns, such as C18 columns. Chaturvedi et al. derivatized HMOs, which were then separated on a C18 column [9]. In addition, analytical methods using mass spectrometry (MS) have been developed with excellent sensitivity and selectivity. However, MS may require additional pretreatment because there is a possibility of device contamination and sensitivity may be reduced by the matrix effect.

Sialyllactose has been previously analyzed in milk primarily using the instruments mentioned earlier. Csernak et al. separated 3′-SL and 6′-SL in human milk within 5 min using a porous graphitic carbon column after solid phase extraction [20]. Santos-Fandila et al. analyzed HMOs containing 3′-SL and 6′-SL in rat serum within 4 min using an amide column by UPLC-MS/MS [21]. In this study, protein precipitation was performed overnight using chloroform and methanol. Fong et al. analyzed milk oligosaccharides containing 3′-SL and 6′-SL in bovine milk within 12 min using a HILIC column following chloroform/methanol extraction [22]. However, there has been no report of the analysis of sialyllactose in organ tissues. We developed an analytical method in minipig tissues using simplified sample preparation steps, as well as a shortened preparation time compared to previous studies. Using a hydrophilic interaction liquid chromatography (HILIC) column, we were able to separate 3′-SL and 6′-SL within 2 min over a relatively short time frame. We adopted a surrogate matrix method to analyze endogenous sialyllactose.

With regard to elimination from the body, the liver and kidney are the major organs involved in the metabolism and excretion of compounds, therefore, the tissue distribution of SL was examined in these organs after intravenous administration. In this study, we developed analytical methods for determination of 3′-SL and 6′-SL in the liver and kidneys of Yucatan minipigs using LC-MS/MS with a simple pretreatment step. The minipig was chosen because it shares anatomical and physiological characteristics with humans, and also has similar biological systems and dietary habits [23].

The surrogate matrix method uses stable isotope-labeled compounds as an internal standard and parallelism was evaluated using this method. The method was validated in accordance with the US Food and Drug Administration (FDA) guidelines [24] and applied to real minipig tissue samples for quantification of SL in the liver and kidneys.

## 2. Results and Discussion

### 2.1. Method Development

The methanol extraction method was simple and sensitive enough to assess sialyllactose in tissues. The methanol extract was evaporated with a SpeedVac concentrator and redissolved with reconstitution solution. The reconstitution solution found to have the best sensitivity and peak shape was water:methanol (40:60, *v/v*).

HILIC column (2.1 × 50 mm, 3 μm; Waters) was selected for 3′-SL and 6′-SL analyses with good separation. The final gradient profile and flow rate were 0.0–1.0 min, 83% B; 1.0–1.1 min, 83–50% B; 1.1–3.0 min, 50% B; 3.0–3.1 min, 50–83% B; 3.1–10 min, 83% B; all at 0.3 mL/min. The aqueous component of the mobile phase for the HILIC column was varied as follows: 0.1% (*v/v*) formic acid in water, 0.1% (*v/v*) acetic acid in water, and 10 mM ammonium acetate buffer (pH 3.5, 4.5, and 5.5, respectively). The organic solvent was acetonitrile. The retention time on the HILIC column decreased somewhat as the pH of the ammonium acetate buffer increased; however, statistical significance was not attained. After considering the resolution, retention time, asymmetry, and sensitivity, as well as the buffer capacity of ammonium acetate (pH 3.8–5.8), 10 mM ammonium acetate buffer (pH 4.5), and acetonitrile were finally selected for the mobile phase. We separated the peaks of 3′-SL and 6′-SL using a HILIC column with a resolution of approximately 2.1 at 1.3 and 1.7 min of retention time, respectively. The peak asymmetry of 3′-SL and 6′-SL was approximately 1.0 and 1.7, respectively. The chromatographic run time per sample was 10 min.

### 2.2. Method Validation

#### 2.2.1. Selectivity

There are three ways to evaluate the selectivity of endogenous compounds using LC-MS/MS [21]. The first method involves setting multiple ion transitions for each analyte and comparing the ratio of the peaks for the multiple ion transitions between standard substances and the authentic matrix. In the second method, the chromatographic conditions, such as gradient profile, mobile phase, or column used, are changed and the retention time of the analyte peak is analyzed for equivalence between the standard substance and the authentic matrix—the analyte peak should not be split into multiple peaks. The third method involves distinguishing between the analyte and its isomer, if available, using the established analytical method.

**Figure 1:**
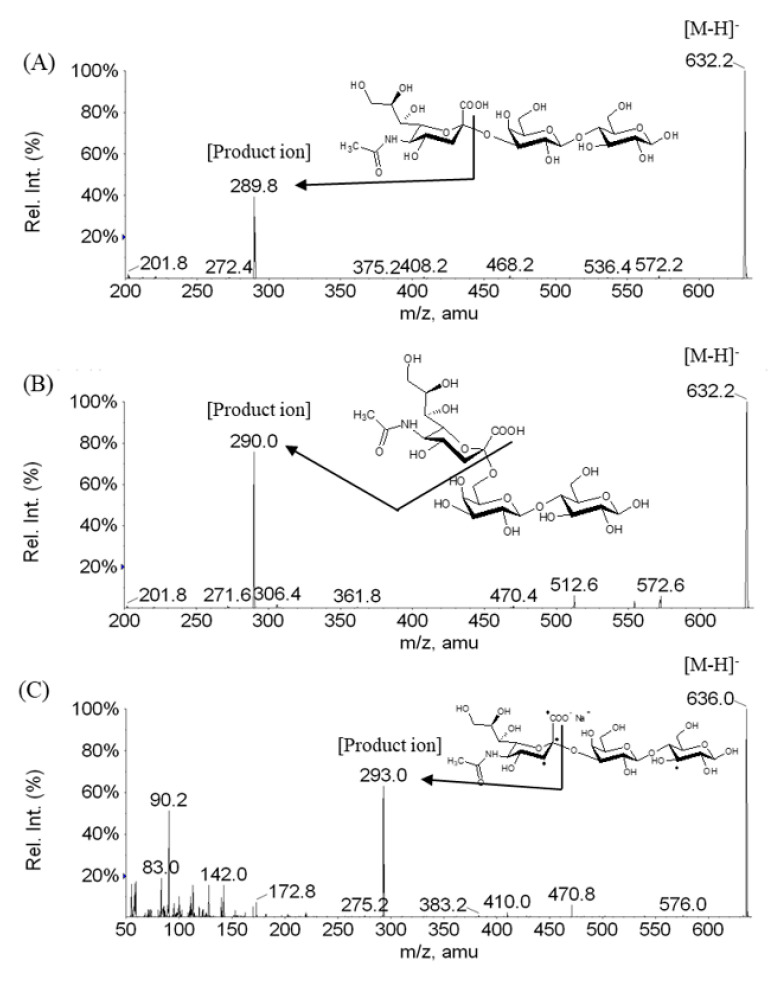
The chemical structure and product ion scan spectra of (**A**) 3′-sialyllactose, (**B**) 6′-sialyllactose, and (**C**) internal standard.

In this study, the first method was used for evaluating selectivity. That is, selectivity was assessed by comparing the peak area ratios of qualified and quantified ion transitions between the HQC in the surrogate matrix and authentic liver and kidney tissue samples. The precursor ion of SL was set to *m/z* 632.4. The product ions were *m/z* 290.0 for quantification and *m/z* 572.2 and *m/z* 469.7 for qualification. The peak area ratios were calculated by dividing the peak area of *m/z* 290.0 by that of *m/z* 572.2 or *m/z* 469.7 (Figure 1).

**Figure 2:**
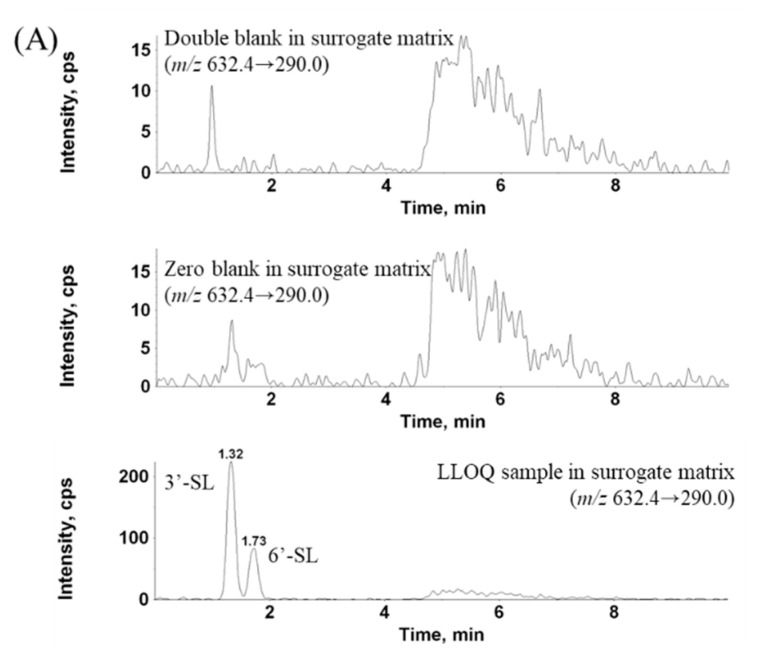

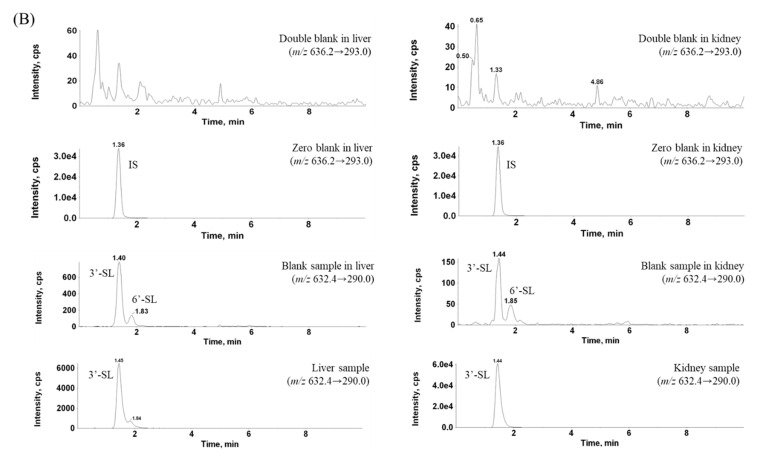
Multiple-reaction monitoring (MRM) chromatograms of (**A**) surrogate matrix and (**B**) liver and kidney: double blank, zero blank, and lower limit of quantification (LLOQ) in surrogate matrix, and double blank, zero blank, blank, and pharmacokinetic application samples for the liver and kidney.

The results indicated no remarkable differences in the peak area ratios of HQC, liver, and kidney between 3′-SL and 6′-SL. The CV (%) of the peak area ratios was 1.7–9.6% for 3′-SL and 6′-SL. The detailed selectivity data are shown in Table 1. No interference was detected with the blank surrogate matrix (water) and standard solution of SL. The LC-MS/MS chromatograms are shown in Figure 2.

#### 2.2.2. Linearity

A calibration curve was generated for the surrogate matrix at concentrations of 20, 40, 500, 1000, 5000, 10,000, and 20,000 ng/g. Linear regression for 3′-SL and 6′-SL was performed with a weighting factor of 1/x^2^, and calculated for each equation and correlation coefficient (r). The r value ranged from 0.9985 to 0.9999, and the accuracy of the mean values in all calibration standard samples ranged from −2.7% to 2.0%, which satisfied the acceptance criteria (within ±15% [±20% for the LLOQ]) of the nominal concentration.

#### 2.2.3. Accuracy and Precision

According to the white paper of Wakamatsu et al. [21], the method for preparing QC samples depends on the concentration of endogenous substances. If the concentration of endogenous compound is lower than the LLOQ, all samples (LLOQ, LQC, MQC, and HQC) are prepared by spiking the standard substance into the authentic matrix. If the concentration of endogenous compound is between the LQC and MQC, then the LLOQ and LQC samples are prepared in a surrogate matrix, or in an authentic matrix diluted with surrogate matrix to ensure a low level of endogenous substance. If the concentration of an endogenous substance is higher than the HQC, QC samples are prepared by diluting the authentic matrix with a surrogate matrix without spiking with the standard. In this study, as the concentration of SL in the tissue was between the LQC (60 ng/g) and MQC (1500 ng/g), LLOQ and LQC samples were prepared in the surrogate matrix, and MQC and HQC samples were prepared in the authentic matrix using blank tissue homogenate.

In liver tissue, the intra-day results for 3′-SL and 6′-SL ranged from −2.8% to 2.7% for accuracy and 0.5% to 7.2% for precision (*n* = 6). The inter-day results for 3′-SL and 6′-SL ranged from −5.3% to 7.5% for accuracy and 2.9% to 10.0% for precision (*n* = 18).

In kidney tissue, the intra-day results for 3′-SL and 6′-SL ranged from −5.3% to 6.0% for accuracy and 1.0% to 7.2% for precision (*n* = 6). The inter-day results for 3′-SL and 6′-SL ranged from −4.7% to 7.5% for accuracy and 3.0% to 10.0% for precision (*n* = 18).

Therefore, 3′-SL and 6′-SL were within the acceptable limits for accuracy and precision according to the FDA guidelines. The precision and accuracy data are summarized in Table 2.

#### 2.2.4. Recovery

Recovery was assessed at the three QC sample concentration levels (60, 1500, and 15,000 ng/g). The results of the recovery test of liver tissue were as follows. The mean recovery ranged from 60.0% to 84.8% for 3′-SL, 59.2% to 85.8% for 6′-SL, and 76.1% to 94.3% for internal standard. Precision (*n* = 6) ranged from 4.7% to 10.1% for 3′-SL, 6.9% to 27.8% for 6′-SL, and 2.0% to 7.9% for internal standard. The results of the recovery test of kidney tissue were as follows. The mean recovery ranged from 90.3% to 123.6% for 3′-SL, 91.2% to 115.7% for 6′-SL, and 95.1% to 96.2% for internal standard. Precision (*n* = 6) ranged from 4.2% to 4.9% for 3′-SL, 2.9% to 14.9% for 6′-SL, and 3.6% to 4.2% for internal standard.

Precision met the acceptance criteria for 3′-SL and 6′-SL, except for 6′-SL in liver tissue (value of 27.8%). This was thought to be due to the background concentration of SL in the authentic matrix being higher than the spiked concentration of LQC. The recovery data are shown in Table 3.

#### 2.2.5. Matrix Effect

The matrix effect was evaluated using six individual authentic samples at the LQC, MQC, and HQC concentration levels of 60, 1500, and 15,000 ng/g, respectively. The results of the matrix effect test of liver tissue were as follows. The mean values of the internal standard-normalized matrix factor for six individual samples ranged from 0.952 to 1.15 for 3′-SL and 1.06 to 1.12 for 6′-SL. Precision (*n* = 6) ranged from 3.6% to 12.4% for 3′-SL and 4.2% to 15.7% for 6′-SL.

The results of the matrix effect test of kidney tissue are as follows. The mean values of internal standard-normalized matrix factor for six individual samples ranged from 0.853 to 0.993 for 3′-SL and 0.889 to 0.996 for 6′-SL. Precision (*n* = 6) ranged from 0.4% to 7.3% for 3′-SL and 1.3% to 20.9% for 6′-SL.

Precision was within the acceptance criteria for 3′-SL and 6′-SL, except for the LQC of 6′-SL for liver (15.7%) and kidney (20.9%). As in the recovery evaluation, this was thought to be because variation in the background concentration of SL in the authentic matrix affects that of the LQC. The results for the matrix effect are shown in Table 3.

#### 2.2.6. Parallelism

The background concentration in the authentic matrix was calculated from the x-intercept of the calibration curve constructed with addition of internal standard to the authentic matrix. The calibration curve equations for 3′-SL and 6′-SL were *y* = 0.000119*x* + 0.0386 and *y* = 0.0000474*x* + 0.00556 in the liver, and *y* = 0.000114*x* + 0.0756 and *y* = 0.0000416*x* + 0.00245 in the kidney, respectively. The background concentration in the authentic matrix was 324 ng/g for 3′-SL and 117 ng/g for 6′-SL in the liver, and 66.3 ng/g for 3′-SL and 58.9 ng/g for 6′-SL in the kidney.

The three QC samples (LQC, MQC, and HQC) in the authentic matrix were analyzed with six replicates and substituted into the calibration curve of the surrogate matrix. Accuracy and precision were assessed by comparing the measured concentrations with the nominal concentrations added to the background concentration in the authentic matrix. The accuracy (RE) of 3′-SL and 6′-SL ranged from −7.2% to −4.6% for the liver and −14.8% to −6.6% for the kidney. Precision (CV, *n* = 6) values for 3′-SL and 6′-SL ranged from 0.4% to 5.6% for the liver and 0.7% to 7.9% for the kidney. The results of the parallelism tests are shown in Table 4.

**Figure 3:**
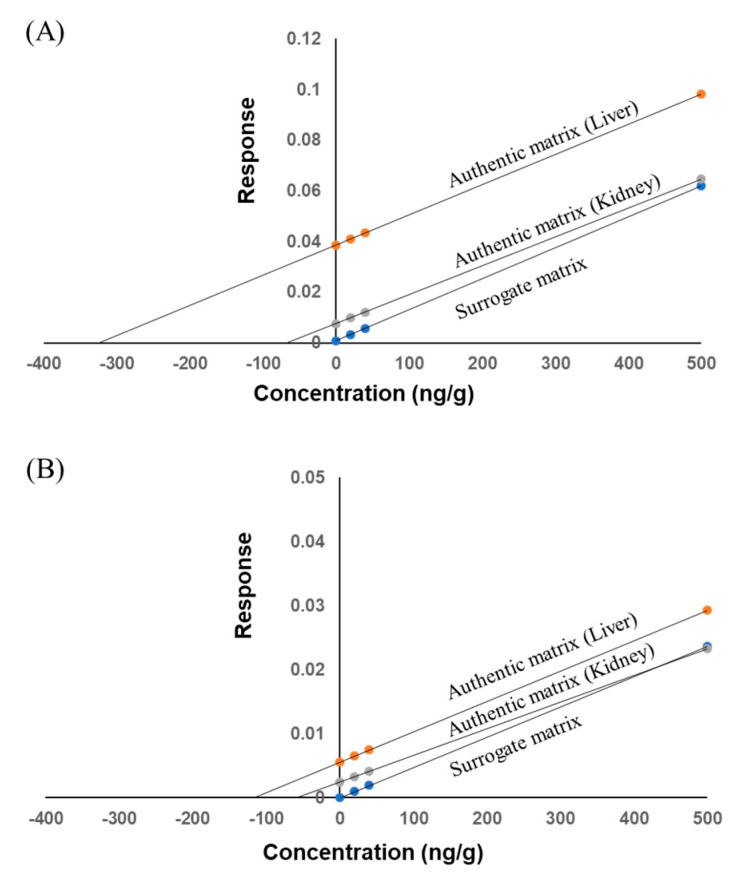
Calibration curves of (**A**) 3′-SL and (**B**) 6′-SL for parallelism assessment in minipig liver and kidney and surrogate matrix. The calibration curves of liver and kidney were constructed by the standard addition method.

In addition, the slopes of the calibration curves for the surrogate and authentic matrix were compared (Figure 3). The slopes for the surrogate matrix and liver tissue were parallel with each other. Although the slopes of the surrogate matrix and kidney tissue were not completely parallel, the accuracy of all QC samples satisfied the acceptance criterion. Thus, we do not believe there are any issues with our quantification.

These results showed that the surrogate matrix can be applied when analyzing minipig tissue using our method.

#### 2.2.7. Carryover

Carryover was assessed by analyzing the ULOQ sample (20,000 ng/g) followed by the blank sample in the surrogate matrix. No peaks were observed at the retention time of 3′-SL and internal standard in the blank sample. The peak area in the blank sample was 2.1% of that in the LLOQ sample at the retention time of 6′-SL. These results satisfied the acceptance criterion, i.e., that the peak area at the retention time of SL should be <20% of that of the LLOQ sample.

#### 2.2.8. Dilution Integrity

If the concentration exceeded the ULOQ, it would be necessary to dilute the sample with blank matrix. Dilution DQCs above the ULOQ were prepared in six replicates in the blank tissue homogenate at a concentration of 100,000 ng/g. Dilution integrity was evaluated based on the accuracy and precision of the samples diluted 10 times with the surrogate matrix (water). The accuracy (RE) was −8.0% for 3′-SL and 12.0% for 6′-SL in the liver, and −9.0% for 3′-SL and 14.0% for 6′-SL in the kidney. Precision (CV, *n* = 6) was 0.6% for 3′-SL and 1.5% for 6′-SL in the liver, and 0.5% for 3′-SL and 1.2% for 6′-SL in the kidney. These results satisfied the ± 15% acceptance criterion, suggesting that samples with concentrations exceeding the ULOQ may be diluted prior to the analysis.

#### 2.2.9. Stability

At the initiation of the stability test, the background concentration in the blank authentic matrix was measured to determine the nominal concentration of the LQC. In the liver tissue, the background concentrations of 3′-SL and 6′-SL were 339 and 118 ng/g, respectively. In the kidney tissue, the background concentrations of 3′-SL and 6′-SL were 62.1 and 55.6 ng/g, respectively.

Benchtop and long-term stability were evaluated simultaneously. The benchtop stability test was carried out at room temperature (24 °C) for 4 h using samples with long-term stability, i.e., those stored at −80 °C for 34 days. All QC samples of 3′-SL and 6′-SL showed stability values of −7.6% to 3.7% for liver and −10.1% to −6.0% for kidney. The post-preparative stability (RE) of samples after 21 h of storage at 10 °C in an autosampler tray ranged from –3.1% to 8.7% for liver QC samples and −10.2% to 4.7% for kidney QC samples of 3′-SL and 6′-SL. To investigate freeze/thaw stability, QC samples were frozen at −80 °C and thawed at room temperature over four cycles. The stability of all QC samples of 3′-SL and 6′-SL ranged from −6.7% to 7.6% for the liver and −9.3% to −3.2% for the kidney. All stability results satisfied the acceptance criterion of ±15% of the nominal concentration. Table 5 summarizes the stability data for the 3′-SL and 6′-SL samples.

### 2.3. Tissue Distribution Study of 3′-SL and 6′-SL in Minipigs

A tissue distribution study was conducted involving intravenous injection of minipigs with 3′-SL and 6′-SL at a dose of 12.5 mg/kg. Kidney, liver, and blood samples were collected. The tissue concentrations and pharmacokinetic parameters are shown in Table 6. The tissue to plasma (T/P) ratio was also calculated from the concentrations of 3′-SL and 6′-SL in the kidney, liver, and plasma, from the same minipigs at the same time points.

The results indicate 3′-SL and 6′-SL were rapidly distributed to the kidney and liver tissue following intravenous gavage. In addition, the T/P ratio of the kidney was 1.0–1.3 fold and that of the liver was 0.12–0.14 fold, indicating that 3′-SL and 6′-SL were shown to be primarily eliminated by the kidney via urine excretion. The exposure of tissues to 6′-SL was approximately 20% higher than that of 3′-SL, with a 6′-SL concentration of 3030 ng/g in the liver and 27,000 ng/g in the kidney. The 3′-SL levels were 2550 ng/g in the liver and 22,300 ng/g in the kidney. However, the difference between 3′-SL and 6′-SL was not found to be based on tissue distribution.

These results provide useful information for further studies on the tissue distribution of 3′-SL and 6′-SL. Additionally, the main excretion route requires elucidation by monitoring the amount of 3′-SL and 6′-SL in bile or urine over time.

## 3. Materials and Methods

### 3.1. Chemicals and Reagents

Standards of 3′-SL (purity 98.55%) and 6′-SL (purity 98.75%) were provided by GeneChem Inc. (Daejeon, Korea). Methanol and acetonitrile (HPLC grade) were purchased from Burdick & Jackson (Muskegon, MI, USA). Acetic acid (HPLC grade) was purchased from Sigma-Aldrich (St. Louis, MO, USA). Ammonium acetate (HPLC grade) was purchased from Fluka (Muskegon, MI, USA). Deionized water was obtained using a Millipore Elix and Milli-Q Biocel system (Millipore, Milford, MA, USA). An internal standard, [1,2,3-^13^C^3^]3′-sialyl[3 -^13^C^glc^] lactose sodium salt, was purchased from Omicron Biomedicals, Inc. (South Bend, IN, USA).

### 3.2. Liquid Chromatography Conditions

Liquid chromatography was performed using a 1200 series system from Agilent Technologies (Santa Clara, CA, USA) comprised of a binary pump, degasser, autosampler and column oven. An Atlantis HILIC column (50 mm × 2.1 mm, 3 μm particle size; Waters, Milford, MA, USA) was used with a mobile phase consisting of (A) 10 mM ammonium acetate in water (pH 4.5) and (B) acetonitrile with gradient elution at a flow rate of 0.3 mL/min. Samples (2 μL) were analyzed using the following gradient profile: 0.0–1.0 min, 83% B; 1.0–1.1 min, 83–50% B; 1.1–3.0 min, 50% B; 3.0–3.1 min, 50–83% B; and 3.1–10 min, 83% B for equilibration to the initial conditions. The column oven and autosampler temperatures were maintained at 40 and 6 °C, respectively.

### 3.3. Mass Spectrometry Conditions

The chromatograph was connected to a triple quadrupole mass spectrometer with an ESI interface. MS was performed using an API4000 instrument (AB Sciex, Framingham, MA, USA) operated in negative ion mode. The ion source parameters were as follows: collision gas (CAD), 6 psi; ion spray voltage (IS), −4500 V; ion source temperature (TEM), 500 °C; nebulizing gas (GS1), 50 psi; and drying gas (GS2), 50 psi. The parameters of the mass spectrometer, including the declustering potential (DP), entrance potential (EP), collision energy (CE), and collision exit potential (CXP) were optimized at −94 V, −10 V, −37 eV, and −7 V, respectively. The ion transitions in multiple-reaction monitoring (MRM) were monitored at *m/z* 632.4→290.0 for both 3′-SL and 6′-SL, and at *m/z* 636.2→293.0 for the internal standard. The data were acquired using Analyst software (version 1.4.2; AB Sciex).

### 3.4. Preparation of Standard Stock Solutions and Homogenized Tissue Samples

Standard stock solutions of 3′-SL and 6′-SL were prepared separately by dissolving each compound in water at a concentration of 1 mg/mL. Working solutions for 3′-SL and 6′-SL were prepared by serially diluting the stock solutions with water. The internal standard was prepared using a stable isotope-substituted sialyllactose [1,2,3-^13^C^3^]3′-Sialyl[3-^13^C^glc^] lactose sodium salt, and the stock solution was prepared by dissolving in water at a concentration of 0.1 mg/mL. Water was used as a surrogate matrix for minipig tissue to prepare the calibration curve and low quality control (LQC). Samples for the calibration curve were prepared by adding 5 μL of suitable working solution to 95 μL of the surrogate matrix, with final concentrations of 20, 40, 500, 1000, 5000, 10,000, and 20,000 ng/g for both 3′-SL and 6′-SL. LQC samples (60 ng/g) were prepared using the surrogate matrix, and middle quality control (MQC; 1500 ng/g) and high quality control (HQC; 15,000 ng/g) samples were prepared using homogenized minipig tissues.

### 3.5. Sample Preparation

Approximately 1 g of minced liver or kidney tissue was weighed and transferred into a polypropylene tube, after which 9 mL of water was added. The mixture was thoroughly homogenized (Powergen 1000; Fisher Scientific, Waltham, MA, USA). Aliquots of 40 μL of the homogenized minipig tissue samples were placed in polypropylene tubes with 40 μL of internal standard solution (1000 ng/mL in water) and 500 μL of methanol. The mixture was then vortexed for 10 min and centrifuged at 16,363 × *g* for 10 min at 10 °C. The supernatant (400 μL) was transferred to fresh polypropylene tubes and evaporated at 45 °C for 90 min using a speed vacuum (miVac Quattro concentrator; Genevac Ltd., Ipswich, UK). The residue was reconstituted with 100 μL of a mixture of 10 mM ammonium acetate in water (pH 4.5) and acetonitrile (mobile phase A and B, 4:6, *v/v*), and vortexed for 10 min. An aliquot of 2 μL was injected into the LC-MS/MS system.

### 3.6. Method Validation

The method developed here was validated in terms of selectivity, linearity, precision, accuracy, the matrix effect, recovery, parallelism, dilution integrity, carryover, and stability according to the FDA guidelines [24].

#### 3.6.1. Selectivity

The selectivity of an analytical method is usually evaluated by comparing the chromatograms of the blank samples from individual sources and standard fortified samples to determine the presence of interference at the retention time of the analyte. However, as SL is an endogenous compound, selectivity was evaluated in a different way. Two qualification ion transitions were established, and the peak area ratios between the qualified and quantified ion transitions of SL were calculated. The ratios of peak area between the HQC sample in the surrogate matrix and the authentic liver and kidney tissue samples were compared and evaluated for equivalence, as described previously [25]. The 3′-SL and 6′-SL isomers were identified by comparing the retention time and ion pair transition between the standard and samples.

#### 3.6.2. Linearity

To prepare a calibration curve, samples were made at seven concentrations in the surrogate matrix and consisted of double blank (without analyte or IS), zero blank (without analyte but with IS), 20, 40, 500, 1000, 5000, 10,000, and 20,000 ng/g. The calibration curve was acceptable when the concentration of each calibrator was within ±15% (±20% for the lower limit of quantification (LLOQ)) of the nominal concentration, and when at least 75% of the calibrator, including the LLOQ and upper limit of quantification (ULOQ), met the acceptance criteria.

#### 3.6.3. Accuracy and Precision

The intra-day and inter-day accuracy and precision were tested at the LLOQ (20 ng/g), and for three QC concentrations (60, 1500, and 15,000 ng/g; LQC, MQC, and HQC, respectively). Accuracy and precision are expressed as the relative error (RE; %) and coefficient of variation (CV; %), respectively. According to the FDA guidelines, accuracy and precision should be within ±15% (±20% for LLOQ) and <15% (20% for LLOQ).

The intra-day test was performed with six replicates at each QC level within 1 day. The inter-day test was performed by repeating the intra-day test for three days. LLOQ and LQC samples were prepared using a surrogate matrix. For the MQC and HQC samples, the background concentration of SL was determined by analyzing the homogenized blank minipig tissue sample, and the background concentration was subtracted from the measured concentrations [25]. The accuracies of the MQC and HQC were calculated by the following equation:

Relative error (%) = (Measured conc. of QC − Background conc.)/Nominal conc. × 100.

#### 3.6.4. Recovery

A recovery test was performed on the three QC samples (60, 1500, and 15,000 ng/g; LQC, MQC, and HQC, respectively) with six replicates per level. Recovery was evaluated by comparing the peak area of a blank sample fortified with a known amount of analyte after sample preparation and the peak area of the sample fortified with the analyte before extraction. The final peak area was calculated by subtracting the peak area of background SL in the authentic matrix. The precision (CV; %) of recovery should be within 15%.

#### 3.6.5. Matrix Effect

The matrix effect refers to the enhancement or suppression of a chromatography signal by interference or co-eluting compounds in the matrix. In this study, the matrix effect was evaluated at 60, 1500, and 15,000 ng/g by an internal standard-normalized matrix factor, which was calculated as the matrix factor of the analyte divided by that of the internal standard. The matrix factor was calculated as the peak area of six individual blank samples fortified with a known analyte after sample extraction divided by that of standard solution. The peak area of analyte in the authentic matrix was calculated by subtracting the peak area of background SL. The precision (CV; %) of the internal standard-normalized matrix factor among six individual samples at each QC should be within 15%.

#### 3.6.6. Parallelism

Parallelism shows that the calibration curve in the authentic matrix is parallel to that in the surrogate matrix. The FDA guidelines specify that parallelism should be evaluated for endogenous compounds [24]. In previous studies, we found methods for evaluating parallelism for endogenous compounds in surrogate matrix or surrogate analyte were described [26,27,28,29], as follows: assessment of precision and accuracy by substituting QCs in authentic matrix into the calibration curve in surrogate matrix; comparison of the calibration curves of authentic and surrogate matrix; and assessment of dilution integrity in authentic matrix. In this study, parallelism was evaluated by the first and second of these methods.

#### 3.6.7. Carryover

The carryover test was performed to evaluate whether the previous sample affects the response of the next sample. Carryover was assessed by analyzing the ULOQ sample followed by a blank sample in the surrogate matrix. In the blank sample, the peak area at the retention time of SL should be <20% of that of the LLOQ sample.

#### 3.6.8. Dilution Integrity

If the determined concentration exceeds ULOQ (20,000 ng/g), the sample should be diluted. In cases with dilution integrity, the diluted sample can be quantified accurately. Six dilution quality control (DQC) samples of 100,000 ng/g were prepared in the homogenized blank tissues and then diluted 10-fold with surrogate matrix. The diluted samples were evaluated for accuracy and precision by substituting them into the calibration curve in surrogate matrix.

#### 3.6.9. Stability

The biological samples were evaluated for benchtop, freeze/thaw, post-preparative, and long-term stability. The stability tests were performed by measuring LQC and HQC samples with three replicates. The background concentration of SL in the blank authentic tissues was set to the nominal LQC concentration in the stability test. As the background concentration of SL in the authentic matrix is similar to or higher than the LQC (60 ng/g), the deviation of the background concentration of SL markedly affects the accuracy of LQC. The background concentration of the blank samples was determined on initiation of the stability test. The HQC samples were prepared by adding a standard solution to the authentic matrix, and the results were calculated by subtracting the endogenous concentration in the blank matrix. Benchtop and long-term stability were evaluated simultaneously with the benchtop stability test carried out at room temperature (approximately 24 °C) for 4 h using samples with long-term stability (stored at −80 °C for 34 days). The post-preparative stability was assessed at 10 °C on an autosampler tray for 21 h. The freeze/thaw stability was evaluated by four cycles of freezing at −80 °C and thawing at room temperature.

### 3.7. Tissue Distribution Study in Minipigs

The tissue distribution of 3′-SL and 6′-SL in Yucatan minipigs was assessed following intravenous administration at a dose of 12.5 mg/kg. The animal experiments were performed in compliance with the Animal Welfare Act and Guide for the Care and Use of Laboratory Animals of Institute for Laboratory Animal Research (ILAR). The animals were kept in a single cage at constant room temperature (23 ± 3 °C) and 30–70% humidity under a regular 12-h light/12-h dark cycle. Animals were provided with approximately 25 g/kg/day food and water was freely available.

Two minipigs were sacrificed at 2 h after administration; liver and kidney tissues, and blood from the jugular vein, were then collected. Each tissue was immediately removed and washed with normal saline, blotted dry with a paper towel, cut into pieces with scissors, and individually homogenized with three volumes of normal saline (*v/w*) in an Ultra-Turrax apparatus (IKA Works, Wilmington, NC, USA). After blood collection into K_2_-EDTA tubes, blood samples were gently mixed and placed on crushed wet-ice/Kryorack (Streck Laboratories, Omaha, NE, USA). Following centrifugation at 20,740 × *g* for 3 min at 4 °C for separation of plasma, the plasma was transferred into labeled polypropylene tubes.

The collected tissues were analyzed using the method developed in this study, and the plasma was analyzed by the method described in our previous study (in press).

The results are presented as the mean ± SD. Statistical analyses were performed using Microsoft Excel 2016 (Microsoft Corp., Redmond, WA, USA) or Phoenix WinNonlin Professional (version 8.1; Certara, Princeton, NJ, USA).

## 4. Conclusions

A very rapid and highly sensitive LC-MS/MS method, for the separation and quantification of 3′-SL and 6′-SL in the liver and kidney tissue of minipigs, was developed and validated in terms of selectivity, linearity, accuracy, precision, matrix effects, recovery, dilution integrity, carryover, and stability. This analytical method, which was developed using the surrogate matrix method (because SL is an endogenous compound) was applied successfully to minipig tissue samples in a tissue distribution study. The developed method was useful for evaluating the tissue distribution of 3′-SL and 6′-SL in the minipig model.

The results showed that 3′-SL and 6′-SL were distributed rapidly to the kidney and liver tissue after intravenous gavage. In addition, 3′-SL and 6′-SL were shown to be mainly eliminated via the kidney with urine excretion. These results provided useful information for further studies for elucidation of main excretion route by monitoring the amount of 3′-SL and 6′-SL.

## Figures and Tables

**Table 1:** Assessment of selectivity for 3′-SL and 6′-SL.

Samples	Peak Area Ratio			
	3′-SL		6′-SL	
	Quant ^a^/Qual1 ^b^	Quant/Qual2 ^c^	Quant/Qual1	Quant/Qual2
HQC ^d^	120	2290	1.83	18.2
Liver	116	2000	1.82	17.3
Kidney	119	2420	1.69	16.3
Mean	119	2240	1.78	20.0
SD ^e^	2	215	0.08	1.0
CV(%) ^f^	1.7	9.6	4.3	4.9

^a^ Quant = The peak area at the quantification ion transition (*m/z* 632.4→290.0). ^b^ Qual1 = The peak area at the first qualification ion transition (*m/z* 632.4→572.2). ^c^ Qual2 = The peak area at the second qualification ion transition (*m/z* 632.4→469.7). ^d^ HQC = high quality control. ^e^ SD = standard deviation. ^f^ CV (%) = standard deviation of the concentration/mean concentration × 100.

**Table 2:** Intra- and inter-day accuracy and precision data.

		3′-SL				6′-SL			
		LLOQ	LQC	MQC	HQC	LLOQ	LQC	MQC	HQC
Nominal Concentration (ng/g)		20	60	1500	15,000	20	60	1500	15,000
(A) Liver									
Intra-day (*n* = 6)	Mean estimated conc. (ng/g)	20.1	59.5	1460	14,900	20.5	58.3	1540	15,300
	RE (%) ^a^	0.3	−0.8	−2.7	−0.7	2.4	−2.8	2.7	2.0
	CV (%) ^b^	3.6	2.7	2.1	0.5	7.2	4.7	1.4	1.7
Inter-day (*n* = 18)	Mean estimated conc. (ng/g)	21.5	59.9	1420	14,300	21.4	59.4	1520	15,400
	RE (%)	7.5	−0.2	−5.3	−4.7	7.2	−0.9	1.3	2.7
	CV (%)	8.5	3.0	4.1	4.9	10.0	4.5	2.9	4.0
(B) Kidney									
Intra-day (*n* = 6)	Mean estimated conc. (ng/g)	20.1	59.5	1470	14,200	20.5	58.3	1590	14,900
	RE (%)	0.3	−0.8	−2.0	−5.3	2.4	−2.8	6.0	−0.7
	CV (%)	3.6	2.7	1.0	3.6	7.2	4.7	2.3	2.6
Inter-day (*n* = 18)	Mean estimated conc. (ng/g)	21.5	59.9	1460	14,300	21.4	59.4	1600	15,400
	RE (%)	7.5	−0.2	−2.7	−4.7	7.2	−0.9	6.7	2.7
	CV (%)	8.5	3.0	3.4	3.8	10.0	4.5	8.3	4.5

^a^ RE (%) = (calculated concentration − theoretical concentration)/theoretical concentration × 100. ^b^ CV (%) = standard deviation for the concentration/mean concentration × 100.

**Table 3:** Matrix effect and recovery data.

		3′-SL			6′-SL		
		LQC	MQC	HQC	LQC	MQC	HQC
Nominal Concentration (ng/g)		60	1500	15,000	60	1500	15,000
(A) Matrix factor (internal standard-normalized, *n* = 6)							
Liver	Mean	1.15	0.952	0.993	1.10	1.06	1.12
	CV (%)	12.4	5.6	3.6	15.7	8.2	4.2
Kidney	Mean	0.853	0.958	0.993	0.889	0.965	0.996
	CV (%)	7.3	1.2	0.4	20.9	2.1	1.3
(B) Recovery (*n* = 6)							
Liver	Mean (%)	84.8	80.5	60.0	85.8	80.8	59.2
	CV (%)	4.7	10.1	7.2	27.8	10.0	6.9
Kidney	Mean (%)	123.6	95.9	90.3	115.7	98.0	91.2
	CV (%)	4.9	4.2	4.4	14.9	2.9	4.3

**Table 4:** Assessment of parallelism between surrogate matrix and authentic matrix.

	3′-SL			6′-SL		
	LQC	MQC	HQC	LQC	MQC	HQC
Nominal Concentration (ng/g)	60	1500	15,000	60	1500	15,000
(A) Liver						
Endogenous conc. (ng/g) ^a^	324	324	324	117	117	117
Adjusted QC conc. (ng/g) ^b^	384	1824	15,324	177	1617	15,117
Mean estimated conc. (ng/g)	357	1701	14,368	168	1543	14,105
RE (%)	−7.2	−6.8	−6.2	−5.5	−4.6	−6.7
CV (%)	1.2	0.6	0.4	5.6	1.9	1.4
(B) Kidney						
Endogenous conc. (ng/g)	66.3	66.3	66.3	58.9	58.9	58.9
Adjusted QC conc. (ng/g)	126	1566	15,066	119	1559	15,059
Mean estimated conc. (ng/g)	108	1445	14,068	108	1392	13,625
RE (%)	−14.8	−7.8	−6.6	−9.5	−10.7	−9.5
CV (%)	6.0	1.3	1.4	7.9	2.5	0.7

^a^ The endogenous concentration in the authentic matrix was calculated from the x-intercept of the calibration curve established with addition of internal standard to authentic matrix. ^b^ The adjusted QC concentration was calculated as the sum of the endogenous concentration and the theoretical concentration of sialyllactose.

**Table 5:** Biological sample stability.

	Liver				Kidney			
	3′-SL		6′-SL		3′-SL		6′-SL	
	LQC	HQC	LQC	HQC	LQC	HQC	LQC	HQC
Nominal Concentration (ng/g) ^a^	339	15,000	118	15,000	62.1	15,000	55.6	15,000
(A) Benchtop (room temperature for 4 h) and long-term stability (storage at −80 °C for 34 days, *n* = 3) ^b^								
Mean estimated concentration (ng/g)	313	14,200	122	14,500	56.4	14,100	50.0	14,000
RE (%)	−7.6	−5.3	3.7	−3.3	−9.2	−6.0	−10.1	−6.7
CV (%)	1.0	0.7	10.5	0.8	11.2	2.7	11.7	2.5
(B) Post-preparative stability at 10 °C for 21 h (*n* = 3)								
Mean estimated concentration (ng/g)	334	14,600	114	16,300	55.7	14,500	55.9	15,700
RE (%)	−1.3	−2.7	−3.1	8.7	−10.2	−3.3	0.5	4.7
CV (%)	2.3	1.0	10.5	0.6	0.7	1.8	10.9	1.6
(C) Freeze/thaw stability (four cycles, *n* = 3)								
Mean estimated concentration (ng/g)	319	140,00	127	14,200	60.1	13,800	51.8	13,600
RE (%)	−5.8	−6.7	7.6	−5.3	−3.2	−8.0	−6.8	−9.3
CV (%)	2.7	0.8	7.7	1.2	5.9	4.4	4.4	4.2

^a^ The nominal concentration of LQC was determined from the background SL concentration in each matrix. ^b^ Benchtop stability was assessed in samples stored at −80 °C for 34 days.

**Table 6:** Tissue to plasma (T/P) concentration ratios of 3′-SL and 6′-SL at 2 h after intravenous administration of a dose of 12.5 mg/kg.

Compounds	Tissue Concentration (ng/g)			T/P Ratio	
	Liver	Kidney	Plasma	Liver/Plasma	Kidney/Plasma
3′-SL	2550	22,300	21,500	0.12	1.0
6′-SL	3030	27,000	21,000	0.14	1.3
